# A Saliency-Based Sparse Representation Method for Point Cloud Simplification

**DOI:** 10.3390/s21134279

**Published:** 2021-06-23

**Authors:** Esmeide Leal, German Sanchez-Torres, John W. Branch-Bedoya, Francisco Abad, Nallig Leal

**Affiliations:** 1 Facultad de Ingenierías, Universidad Autónoma del Caribe, Barranquilla 080001, Colombia; esleal@uac.edu.co (E.L.); nleal@uac.edu.co (N.L.); 2 Facultad de Ingenierías, Universidad del Magdalena, Santa Marta 470004, Colombia; 3 Facultad de Minas, Universidad Nacional de Colombia-Sede Medellín, Medellín 050041, Colombia; jwbranch@unal.edu.co; 4 Instituto Universitario de Automática e Informática Industrial, Universitat Politècnica de València, 46022 Valencia, Spain; fjabad@dsic.upv.es

**Keywords:** point cloud simplification, sparse representation, saliency features

## Abstract

High-resolution 3D scanning devices produce high-density point clouds, which require a large capacity of storage and time-consuming processing algorithms. In order to reduce both needs, it is common to apply surface simplification algorithms as a preprocessing stage. The goal of point cloud simplification algorithms is to reduce the volume of data while preserving the most relevant features of the original point cloud. In this paper, we present a new point cloud feature-preserving simplification algorithm. We use a global approach to detect saliencies on a given point cloud. Our method estimates a feature vector for each point in the cloud. The components of the feature vector are the normal vector coordinates, the point coordinates, and the surface curvature at each point. Feature vectors are used as basis signals to carry out a dictionary learning process, producing a trained dictionary. We perform the corresponding sparse coding process to produce a sparse matrix. To detect the saliencies, the proposed method uses two measures, the first of which takes into account the quantity of nonzero elements in each column vector of the sparse matrix and the second the reconstruction error of each signal. These measures are then combined to produce the final saliency value for each point in the cloud. Next, we proceed with the simplification of the point cloud, guided by the detected saliency and using the saliency values of each point as a dynamic clusterization radius. We validate the proposed method by comparing it with a set of state-of-the-art methods, demonstrating the effectiveness of the simplification method.

## 1. Introduction

Point clouds have become a standard data input tool for many fields, including scientific visualization, photogrammetry, and medical applications. For data acquisition of 3D shapes, modern 3D scanning devices can produce a vast amount of data, reaching millions of points [1]. This amount of data creates challenges on several fronts, like large storage requirements and increased data transmission and rendering times. To reduce the complexity of such point clouds and make the subsequent geometric processing algorithms more efficient, it is common to simplify the point cloud.

The main requirement for point cloud simplification algorithms is that they should maintain the global shape, the sharp features, and the curvatures of the original cloud. For the last of these, transitions between planar and curved areas should be preserved [2]. It is important to preserve the representative points and the sampling density in order to approximate faithfully the original point cloud both geometrically and topologically. The simplified point cloud must be dense around the sharp features (corners, edges, and curvatures) to preserve the global topology and sparse in flattened regions (low or zero curvature).

Some of the limitations of current simplification algorithms are nonuniformity in the simplified point clouds [3,4], problems in keeping the balance between preserved and lost features [5], reduced accuracy, and high computational cost [6]. Some of the proposed algorithms solve those shortcomings using parameters for tuning the final metric by means of weights of scales, but the burden is on the user to obtain satisfactory results [7]. Other methods present high computational cost because they use clustering algorithms in their initial stages [5,8] and some use only one feature (e.g., normal or curvature) for the simplification [9,10].

In this paper, we propose a reliable, robust, and simple solution for the above problems. Our method uses the normal vector, the surface variation (curvature), and the point coordinates, integrated into a unique feature vector, as input to train a dictionary. There are two advantages of using this approach: on one hand, it is possible to unify different descriptors in a unique feature vector, and on the other hand, it is possible to capture the local and the global structure of the point cloud using dictionary learning and sparse coding representation.

Since sharp features are often sparse, the use of sparsity-based modeling to describe and preserve sharp features is an attractive tool for point cloud simplification.

The main contribution of our work is to use the sparse matrix to analyze the structure of point sets, gathering evidence from local geometry to infer global properties about the objects. When the point cloud sparse matrix representation is very sparse, it means that it has found the intrinsic structure of the input point cloud. In the context of point cloud simplification, this means that the model can properly represent the sampling points, preserving the sharp features and at the same time maintaining the uniformity of the point cloud.

The original point cloud data only contains the coordinates of the points with no topological information. To extract the implicit geometric information (normal vectors, surface variation, curvatures), the point-based simplification algorithms use the local information around each point in the cloud.

Usually, the k-nearest neighbor algorithm is used to estimate such geometric information. For each point in the cloud, the proposed method uses the coordinates of the normal vector, the coordinates of the point, and the curvature as a feature vector to identify potential saliency points. The feature vectors of each data point are the training signals for a dictionary learning process. With the dictionary trained, a sparse coding process is carried out to identify the most salient regions in the point cloud. Finally, the proposed method simplifies the point cloud by using the sparse vectors as a clusterization radius.

Formally, the problem of point cloud simplification is defined as follows: Given a surface S defined by a point cloud P and a target sampling rate N<|P|, the goal is to find a point cloud $P^{\prime }$ with |$P^{\prime }$| = N such that the distance *ε* of the surface $S^{\prime }$ to the original surface S is minimal [6]. Symbolically we write the above as follows:

$$P\to P^{\prime },$$

where $\left|P^{\prime }\right|=N<\left|P\right|$ and $\|P-P^{\prime }\|<\varepsilon$, where |·| is the point cloud cardinality and ‖.‖ is the Euclidean distance. The error limit *ε* is used to enforce that that no point in the simplified cloud $P^{\prime }$ is further than *ε* with respect to the original model.

As far as we know, we have not found in the state of the art any method that uses dictionary learning and sparse coding as a basis for point cloud simplification. The proposed method does not introduce a new technique or modification to the classic dictionary learning and sparse coding algorithms.

The contributions of this paper are as follows:

1. The proposed point cloud simplification method based on dictionary learning and sparse coding maintains a balance between sharp features and the density of point distribution.
2. The proposed method reduces the cardinality of the point cloud very efficiently due to its inherent perceptual nature, which selects important points based on their saliency.
3. The saliency-based simplification provides an importance criterion to preserve the most important geometric features.
4. The analysis of the dispersion matrix $\|\alpha {\|}_{1}$ together with the fit or approximation error $\|x-D\alpha {\|}_{2}^{2}$ (Equation (3)) can be used to determine when a point is salient or not.

## 2. Related Work

In recent decades, a considerable amount of research has been conducted on point cloud simplification. Point cloud simplification algorithms can be roughly divided into four categories: particle simulation-based methods, iteration-based methods, formulation-based methods, and clustering-based methods.

### 2.1. Particle Simulation-Based Methods

Pauly et al. [9] presented a particle simulation method. The proposed algorithm distributes a set of points called particles evenly onto a surface, producing point clouds with low approximation error to the original point cloud. Collections of particle simulation-based methods are called local optimal projection (LOP)-based methods [3]. These methods project a set of points over an underlying surface using a localized version of the L1 median filter regularized by a repulsion potential. Huang et al. [5] proposed a correction over the original LOP algorithm, distributing the points evenly over the underlying surface. Huang et al. [6] and Liao et al. [11] aimed to integrate the vector normal to each projected point in order to preserve sharp features in the point cloud. These methods produce good results for surface simplification but are computationally expensive. Furthermore, the original points are replaced by the particles, changing their location in the process.

### 2.2. Clustering-Based Methods

These methods divide the point cloud into clusters, applying some criteria and then replacing the cluster points with a centroid. Pauly et al. [9] presented two algorithms: uniform incremental clustering and hierarchical clustering. These methods are memory- and time-efficient but produce high average approximation errors with respect to the original surface. Shi et al. [10] presented an adaptive method for simplifying point clouds. They applied a recursive subdivision scheme in which the algorithm selects representative points and removes redundant ones. They used k-means clustering to group similar spatial points and applied the maximum normal vector deviation measure to subdivide the clusters. The algorithm can handle boundaries and produce uniform density in flat regions and high density in curved regions. Mahdaoui et al. [12] presented a comparison between two simplification algorithms using k-means and fuzzy c-means algorithms. The method proposes using a metric based on entropy estimation for clustering the point cloud. Liu et al. [13] presented an edge-sensitive feature detail preserving algorithm; they used two clustering schemas to split the point cloud into the geometric and spatial domains. These methods can preserve global structures of the point clouds, and some of them preserve sharp features; however, because of the clustering process, they are computational time-consuming.

### 2.3. Formulation-Based Methods

These methods are based on mathematically modeled optimality. Leal et al. [8] proposed a three-step method. In the first step, they apply a clusterization algorithm. The second step involves the identification of points with high curvature to be preserved. The last step uses a linear programming model to simplify the point cloud, maintaining a density equivalent to the original point cloud. Chen et al. [14] employed a resampling strategy based on a graph that selects representative points while preserving features. The minimization of the point cloud is carried out by a proposed reconstruction error based on a feature extraction operator. Qi et al. [15] proposed an optimization strategy for maintaining the balance between finding the sharp features and preserving the density in the point cloud. The optimization is represented using a graph filter. The results of this method are superior to some other state-of-the-art methods, but it is computationally expensive.

### 2.4. Iteration-Based Methods

Pauly et al. [9] proposed an iterative simplification method using quadric error metrics. The algorithm produces point clouds with low approximation errors, but they are expensive to compute. Alexa et al. [4] proposed a decimation process based on the moving least square (MLS) method. The proposed method removes redundant information using a surface error metric. The global result of the algorithm is good, but it can produce uneven sampling because the subsampling unnecessarily restricts the potential sampling position. Zang et al. [16] presented a method based on a multilevel strategy for point cloud simplification, which adaptively determines the optimal level of each point. For each level, the method extracts the points based on a measure of importance given by a 3D Gaussian method. Zhu et al. [17] proposed a multiview method for point cloud simplification, projecting the points onto the three orthographic planes, in order to identify the model edges. The edges are merged to produce the 3D edges of the model, and the points with less importance are separated from the point cloud. Shoaib et al. [18] proposed a method called fractal bubble to simplify point clouds, selecting important data points through the expansion of a recursive generation of self-similar 2D bubbles until contact is made with a point. Ji et al. [7] presented a detailed feature points simplified algorithm (DFPSA). They proposed estimating the importance of each point using a four characteristic operator, which involves estimating normal curvature distance between the points and the projection distance to each point in the point cloud. Finally, a threshold is used to decide whether each point may be classified as a feature point or not. The nonfeature points are simplified using an octree structure to avoid creating regions with holes. Zhang et al. [19] presented a feature-preserved point cloud simplification (FPPS) method. For the simplification, an entropy measure is defined, which quantifies the geometric features hidden in the point cloud. Then, the key points are selected based on the entropy.

## 3. Dictionary Learning and Sparse Coding

Dictionary learning is a technique whose goal is to learn a set of overcomplete basis (dictionary) in order to model data vectors as a sparse linear combination of basis elements (atoms of the dictionary) [20].

Formally, the dictionary learning problem can be formulated as follows:

Given a set of training data vectors $X={\left\{x_{i}\in R^{N}\right\}}_{i=1,2,\ldots ,N}$, the aim is to find a basis vector $D={\left\{d_{i}\in R^{N}\right\}}_{i=1,2,\ldots ,N}$, which can sparsely represent the training data vectors in the set X, with α being its sparsest representation. The goal is to minimize Equation (1).

(1) $$\left\{\hat{D},\hat{\alpha }\right\}=\arg\underset{D,\alpha }{\min}\|X-D\alpha {\|}_{2}^{2}s.t.\|\alpha {\|}_{0}\leq L$$

L controls the sparsity of X in D. Equation (1) is minimized using the K-SVD algorithm proposed by Aharon et al. [21].

The purpose of sparse coding [22,23] is to approximate a feature input vector as a linear combination of basis vectors, which are selected from a dictionary that has been learned from the data directly.

Formally, let x be a signal of dimension n; the sparse coding aims to find a dictionary $D=\left\{d_{1},d_{2},\ldots ,d_{N}\right\}$, such that x may be approximated by a linear combination of atoms ${\left\{d_{i}\right\}}_{i=1}^{N}$. This is $x\approx D\alpha =\sum_{j=1}^{N}\alpha_{j}d_{j}$, where most of the coefficients $\alpha_{j}$ are zero or close to zero [20]. Thus, the sparse coding problem can typically be formulated as an optimization problem:

(2) $$\hat{\alpha }=\arg\underset{\alpha }{\min}\|x-D\alpha {\|}_{2}^{2}s.t.\|\alpha_{0}\leq L$$

In this formulation, the dictionary D is given and L once again controls the sparsity of *x* in D. The term $\|\alpha {\|}_{0}$ measures the dispersion of the decomposition and can be understood as the number of nonzero coefficients in α, or sparse coefficients, in order to approximate the signal x as sparsely as possible. Or, alternatively,

(3) $$\hat{\alpha }=\arg\underset{\alpha }{\min}\|x-D\alpha {\|}_{2}^{2}+\lambda \|\alpha {\|}_{1}$$

Equation (3) is an optimization problem where the norm $l_{0}\;\left(\|\cdot {\|}_{0}\right)$ is changed by the norm $l_{1}$ ($\left\|\cdot {\|}_{1}\right)$ and λ is the regularization parameter. The solution to Equation (2) with $l_{0}$ norm is an NP-hard problem; fortunately, under certain conditions, it is possible to relax the problem using $l_{1}$ norm and find an approximated solution using Equation (3) with $l_{1}$ norm.

## 4. Proposed Method

**Figure 1:**
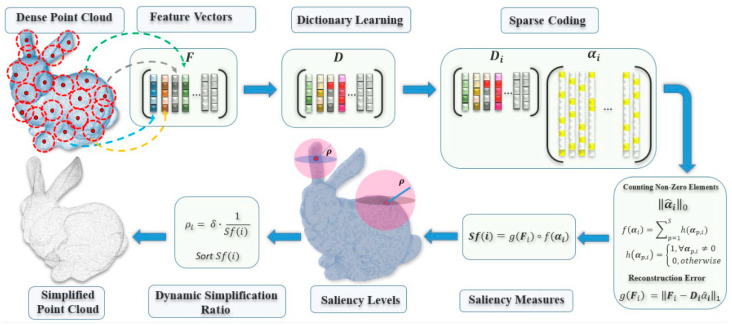
Illustration of the steps involved in the proposed method to simplify a point cloud.

Our proposed method is based on dictionary learning and sparse coding. The input point set is analyzed using the covariance matrix to extract the local features; then, using the dictionary and the sparse representation matrix, the point set is analyzed globally to identify saliency features. Finally, we use the saliencies to sample the point cloud, keeping the most representative points. Figure 1 shows the pipeline of the proposed method.

### 4.1. Selecting the Features

To characterize the point set, we define a descriptor for each point $p_{i}$. The point descriptor is composed of the normal vector, the total variation of surface (curvature), and the point coordinate. With these features, we build a feature vector for each point to measure its importance with respect to the entire set.

The normal vector is used for two reasons. The first is because it can help to identify feature points. A large difference between the normals around a point means that the surface at the point is not planar; that is, it is likely to be a feature point. The second reason is related to the problem of obtaining a simplified point cloud that, when rendered, looks like or mimics the original point cloud from which it was derived. The normals are used in the rendering process to estimate shading and lighting. Therefore, when a point is in a sharp feature, it is considered an important point, and its normal vector must be retained in the simplified point cloud. We use the normal coordinates as components of the feature vector.

The surface curvature captures the surface variation at a point. The curvature is used in several algorithms of point cloud simplification because it is an intrinsic property that intuitively reflects the sharpness of a point in a surface. High curvatures reflect large variations of the surface at the point and hence pinpoint a sharp feature. Therefore, we use the surface variation at the point as a curvature measure, and we include it as a component of the feature vector.

In addition to the normal vector and the surface variation or curvature, the position of each point is also considered in order to guarantee a minimum sample density in every region of the cloud. Without this information, low-saliency areas could be heavily decimated, appearing holes in the point cloud and thus compromising the continuity of the surface when the cloud is rendered. Hence, the coordinate of each point is also used as a component in its feature vector.

### 4.2. Low-Level Feature Estimation

A common way to estimate low-level features in a point set is to apply the principal component analysis (PCA) method locally to each neighborhood around each point $p_{i}$ [9]. Specifically, we use a weighted version of PCA [24,25] with a covariance matrix $Cm_{i}$, as defined in Equation (4).

(4) $$Cm_{i}=\frac{1}{k_{i}-1}\overset{k_{i}}{\underset{j=1}{\sum }}w_{j}\left(p_{j}-\bar{p}\right){\left(p_{j}-\bar{p}\right)}^{T}$$

(5) $$\bar{p}=\frac{1}{k_{i}}\overset{k_{i}}{\underset{j=1}{\sum }}p_{j}$$

where $k_{i}=\left|N_{g}\left(p_{i}\right)\right|$ is the cardinality of the neighborhood around $p_{i}$, $N_{g}\left(p_{i}\right)$; $w_{j}$ is a weight estimated by $w_{j}=exp\left(-\frac{d^{2}}{k_{i}^{2}}\right);\;d=\left|\left|p_{i}-\bar{p}\right|\right|$ is the Euclidean distance. Next, we analyze the eigenvalues $\lambda_{0}\leq \lambda_{1}\leq \lambda_{2}$ and eigenvectors $v_{0},v_{1},v_{2}$ of the covariance matrix $Cm_{i}$.

The eigenvector $v_{0}$ corresponding to the smallest eigenvalue $\lambda_{0}$ is the normal vector $n_{i}$ at point $p_{i}$. Pauly et al. [9,26] proved that the surface variation is equivalent to the surface curvature, as defined in Equation (6).

(6) $$\sigma \left(p_{i}\right)=\lambda_{0}/\left(\lambda_{0}+\lambda_{1}+\lambda_{2}\right)$$

(7) $$n_{i}=\left(n_{x},n_{y},n_{z}\right)$$

(8) $$p_{i}=\left(p_{x},p_{y},p_{z}\right)$$

Once the low-level features are defined, we build a seven-dimensional feature vector $F_{i}$ for each point $p_{i}\in P$, where

$$F_{i}=\left(n_{x},n_{y},n_{z},\sigma ,p_{x},p_{y},p_{z}\right)$$

### 4.3. Dictionary Construction and Sparse Model

Using the feature vectors defined in the above section as data vectors $F_{i}\in R^{n\times 1}$, with n=7 (number of low-level features), we construct the data matrix $F=\left\{F_{1},F_{2},\ldots ,F_{K}\right\}\in R^{n\times K}$, where K=|P| is the number of feature vectors. A sparse coding matrix $\alpha \in R^{S\times K}$ and a dictionary $D\in R^{n\times S}$ are defined using sparse coding theory. S is the number of atoms of the dictionary. Un our experiment, we set S=200; for all the models, the fixed value of the dictionary with S=200 was selected using the mean square error variation. We found that for values greater than 200 atoms, the MSE tends to converge, as is verified in Section 5. The dictionary learning problem is solved using the K-SVD algorithm, as per Aharon et al. [21], obtaining the estimation of α and D. Now F can be reconstructed as F=Dα, obtaining the sparse representation of the data matrix F in the dictionary D. The saliency points can be found by analyzing the sparse matrix α.

### 4.4. Detecting Saliency Points

Once the sparse coding matrix α has been obtained, we analyze what vectors correspond to saliencies. Let $\alpha_{j}$ and $F_{j}$ be column vectors of the matrices α and F, respectively. A feature vector is considered salient if its sparse representation $\|\alpha_{j}{\|}_{1}$ has many nonzero elements—implying that a linear combination of many atoms is required to represent the point correctly—and if its sparse reconstruction error $\|F_{j}-D\alpha_{j}{\|}_{2}$ produces a high residual. On the other hand, a feature vector is not considered salient if its sparse representation $\|\alpha_{j}{\|}_{1}$ has few nonzero elements, i.e., if it can be represented by the linear combination of only a few atoms and its sparse reconstruction error $\|F_{j}-D\alpha_{j}{\|}_{2}$ produces a low residual.

On this basis, we sum the nonzero elements of each column of the matrix α. A score vector with these sums is built as follows:

(9) $$f\left(\alpha_{j}\right)=\sum_{p=1}^{S}h(\alpha_{p,j})\;\forall_{j}=1,2,\ldots ,S$$

(10) $$h\left(\alpha_{p,j}\right)=\{\begin{array}{c} 1,\;\forall \alpha_{p,j}\neq 0\\ 0,\;otherwise \end{array}$$

The sparse reconstruction error is computed by summing the residuals resulting from the difference between each signal $F_{j}$ and its respective reconstruction $D\alpha_{j}$; i.e., $r_{j}=\|F_{j}-D\alpha_{j}{\|}_{2}$. The score vector is defined as follows:

(11) $$g\left(F_{j}\right)=r_{j}\;\forall_{j}=1,2,\ldots ,S$$

Now we normalize the score vectors $f\left(\alpha_{j}\right)$ and $g\left(F_{j}\right)$, dividing each vector by its highest component.

(12) $$f^{\prime }\left(\alpha_{j}\right)=f\left(\alpha_{j}\right)/max\left(f\right)\;\forall_{j}=1,2,\ldots ,S$$

(13) $$g^{\prime }\left(F_{j}\right)=g\left(F_{j}\right)/\max\left(g\right)\;\forall_{j}=1,2,\ldots ,S$$

Next, both score vectors are integrated into a unique score vector as follows:

(14) $$Sf\left(i\right)=f^{\prime }\left(\alpha_{i}\right)g^{\prime }\left(F_{i}\right)\;\forall_{i}=1,2,\ldots ,S$$

**Figure 2:**
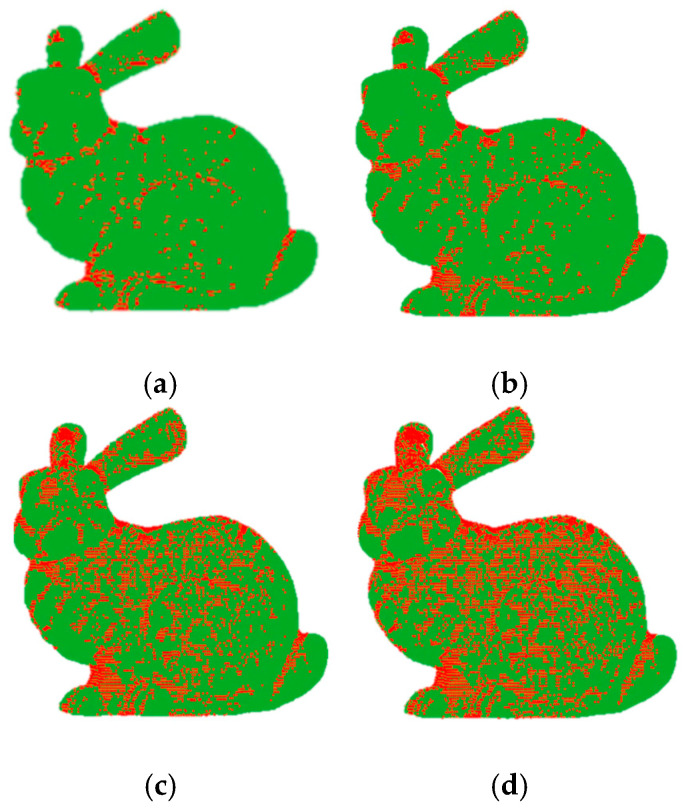
Different levels of saliency produced by the thresholding of the vector Sf with different values: (**a**) T=0.9, (**b**) T=0.8, (**c**) T=0.7 and (**d**) T=0.6.

We use the vector score Sf as a metric for the simplification process. Figure 2 shows the saliency levels found in the vector Sf; to visualize it, we use a threshold T with different values. Equation (14), was proposed by [27] in a local context, and the present work is a generalization to use it globally.

### 4.5. Simplification-Based Saliency

**Figure 3:**
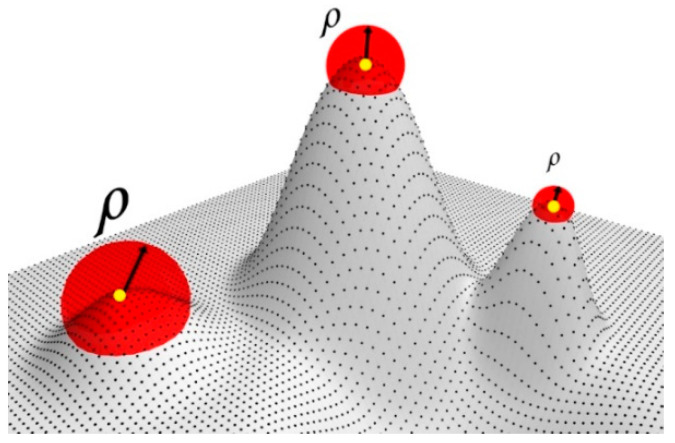
Dynamic ratio.

The saliency points characterize the most relevant features in the point cloud. These points must be retained in the simplification process. On the other hand, points with low saliency are redundant and have less importance for representing the original surface. Using the vector score defined by (14), we establish a dynamic ratio of influence that depends on the importance of the saliency of each point in the entire cloud. If point $p_{i}$ is salient, the ratio of influence will be small, and few points will be removed. If, however, it is not salient, the ratio of influence will be large, and more points will be removed (see Figure 3).

To proceed with the simplification, as a first step, the vector score Sf(i) is sorted by the absolute value of its components. In the second step, we calculate the ratio of influence as follows:

(15) $$\rho_{i}=\delta \cdot \frac{1}{Sf\left(i\right)}$$

According to (15), the dynamic ratio $\rho_{i}$ is determined by 1/Sf(i). Therefore, in points with high saliency, the ratio is small, while in points with low saliency, the ratio is large, as shown in Figure 3, where δ is a user-defined scale parameter that controls the number of points to be simplified.

## 5. Results and Discussion

We evaluated the proposed method using a set of models, namely the Max Planck data set (50,112 points, few detail features), the Fandisk data set (6475 points; high, sharp features), the Asian dragon data set (3,609,600 points, many detail features), the Bunny data set (35,947 points, few detail features), the Elephant data set (24,955 points, many detail features), the Horse data set (48,485 points, few detail features), the Gargoyle data set (25,038 points, many detail features), and the Nicolo data set (50,419 points, few detail features).

We also compared the results of our method to other approaches. For quantitative comparison, our method, which we named saliency dictionary-based simplification (SDBS), is compared to three point-based methods, namely the curvature-based method (CV), implemented using Geomagic Studio; simplification on graph (FPUC) [15]; and fast resampling via graphs (FRGR) [14], and one mesh-based method, namely poisson sampled disk (PSD), implemented using MeshLab. For visual comparison, we replicated the same experiment carried out in [7], and we used the results to compare the proposed algorithm with our method and six state-of-the-art simplification methods: grid simplification (GRID) from CGAL library, hierarchical clustering simplification (HCS) [9], weighted LOP (WLOP) [5], simplification on graph (FPUC) [15], fast resampling via graphs (FRGR) [14] and detailed feature points simplified algorithm (DFPSA) [7].

All the experiments were run on a PC with Intel Core i7-2670QM CPU 2.20 GHz and 8 GB RAM. For implementing the proposed method, we used the MATLAB R2016b programming environment.

**Figure 4:**
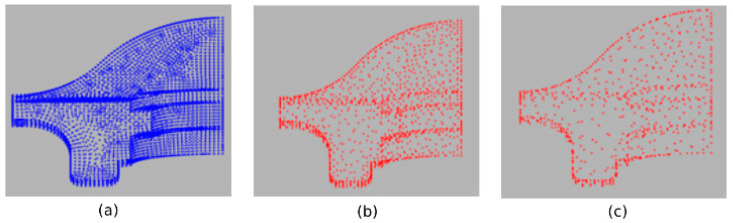
The Fandisk model: (**a**) original 6475 points; (**b**) simplified to 1465 points; (**c**) simplified to 738 points.

**Figure 5:**
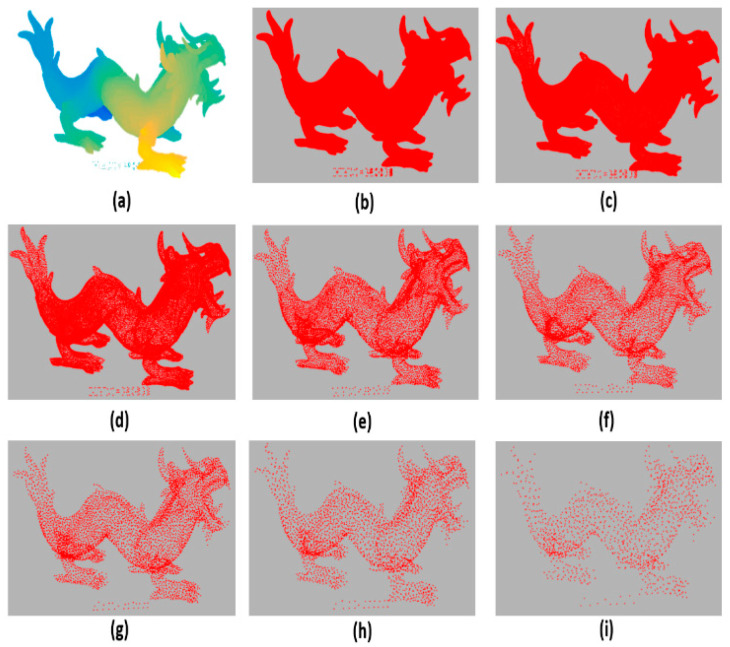
The Asian dragon model: (**a**) original 3,609,600 points; simplified to (**b**) 410,208 points, (**c**) 78,268 points, (**d**) 30,487 points (**e**) 12,621 points (**f**) 8196 points, (**g**) 5758 points, (**h**) 3307 points, and (**i**) 1502 points.

**Figure 6:**
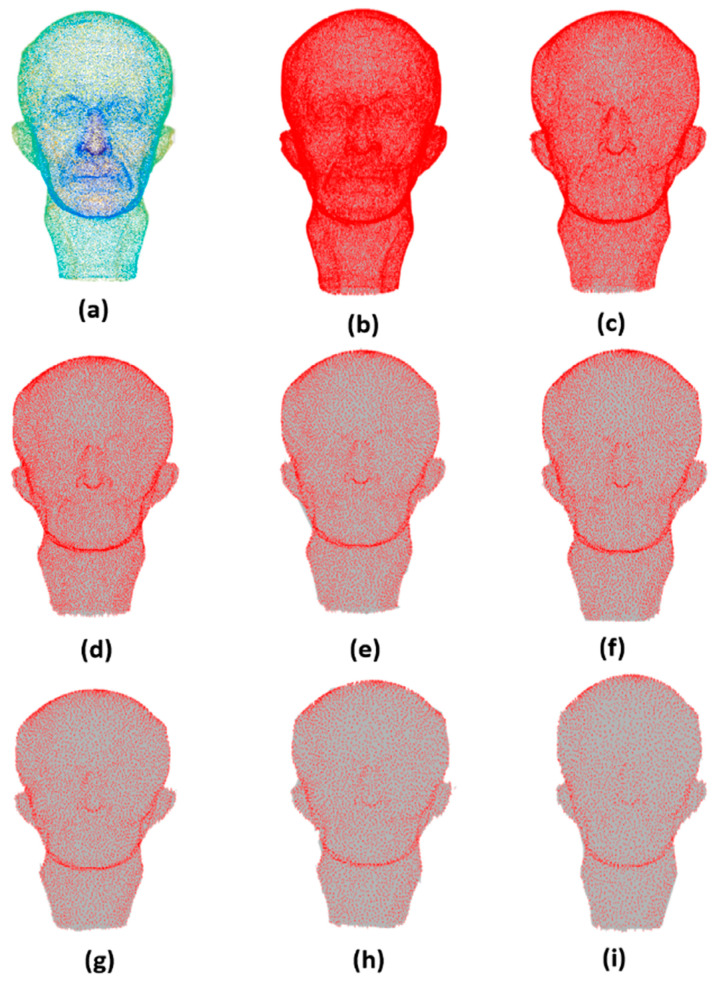
The Max Plank model: (**a**) original 50,112 points; simplified to (**b**) 40,108 points, (**c**) 26,387 points, (**d**) 20,105 points (**e**) 12,761 points (**f**) 8898 points, (**g**) 6588 points, (**h**) 5108 points, and (**i**) 4100 points.

**Figure 7:**
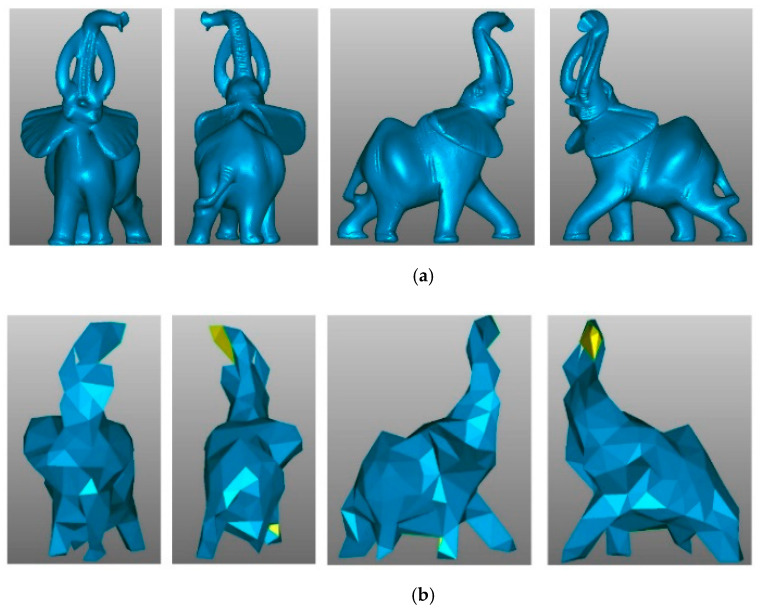
The Elephant model: (**a**) original, reconstructed with 24,955 points; (**b**) simplified, reconstructed with 167 points.

Figure 4, Figure 5, Figure 6 and Figure 7 are examples of the effectiveness of the proposed simplification method in different types of point clouds (free-form surfaces and surfaces with sharp edges and corners). It is clear that the proposed method is capable of preserving the global structure of the clouds as the simplification rate increases in all cloud types, since the needed information is integrated into the dictionary training.

Figure 4 shows the Fandisk model. The edges and corners are preserved as the simplification rate increases, and in flat regions, the method tries to distribute the points evenly.

Figure 5 shows how the Asian dragon model is simplified from millions of points (3,609,600) to thousands (1502). The proposed method preserves the global structure and the most relevant details of the original point cloud.

In Figure 6, it can be appreciated how the Max Plank model is simplified from 50,112 to 1502 points. The proposed method preserves the global structure and some of the details of the original point set. The Max Plank model is a free-form surface, showing that our method operates efficiently over these types of models.

Figure 7 shows the Elephant model simplified from 24,955 to 167 points. The renderings of the simplified and original models are shown from different points of view, showing how the global structure is preserved even with a low sampling rate.

### 5.1. Parameter Selection

**Figure 8:**
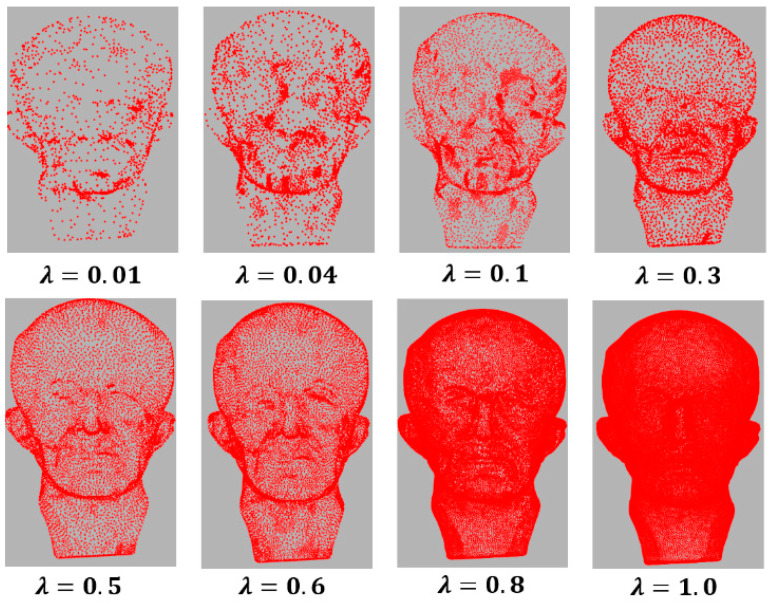
Variation of the parameter λ with the parameter δ=0.36 fixed.

There are three parameters in our method: the regularization parameter λ in Equation (3), the dictionary size S, and the fraction of points to be simplified δ. The parameter λ is the balance between the data fidelity and the regularization term. Small values can produce a simplification with few details, points, and features, while large values can result in more details, points, and features (see Figure 8). In all our tests, we set λ=0.5, which obtains the best results since this value maintains the balance between the number of points and the features.

We established the size of the dictionary, S, based on Figure 8. It shows the mean square error (MSE) variation as the dictionary size increases. As the size of the dictionary increases, the MSE decreases, but processing time increases. On the other hand, when the dictionary size is reduced, the MSE increases, but the processing time decreases. Our goal was to find a balance between a suitable dictionary size and low processing time.

**Figure 9:**
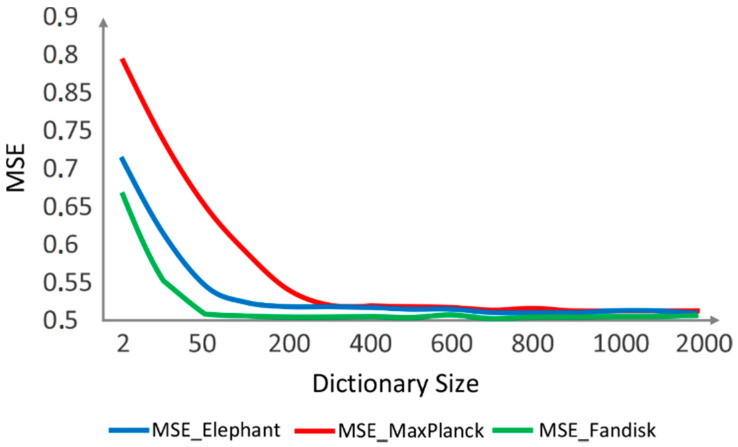
MSE variation vs. dictionary size.

Figure 9 shows that in the range of values between 200 and 400, the MSEs are low, and the size of the dictionary is not significant. In all the experiments, we set the dictionary size S=200, producing good results.

The scale parameter δ is the only free user-defined parameter, and it is used for tuning the number of points to be removed.

### 5.2. Quantitative Analysis Parameter Selection

We chose the geometric error between the original and the simplified point cloud as a metric to evaluate the quality of the proposed simplification method, following Pauly et al. [9]. Similarly, we measured the maximum error distance and the average error distance between the original point cloud, P, and the simplified point cloud, $P^{\prime }$. We denote the surface of P as S and the surface of $P^{\prime }$ as $S^{\prime }$. The simplified error is estimated using the maximum error (16) and the average error (17) as follows:

(16) $$\Delta_{max}\left(S,S^{\prime }\right)=\underset{p_{i}\in S}{\max}\left|d\left(p_{i},S^{\prime }\right)\right|$$

(17) $$\Delta_{avg}\left(S,S^{\prime }\right)=\frac{1}{\left\|S\right\|}\underset{p_{i}\in S}{\sum }\left|d\left(p_{i},S^{\prime }\right)\right|$$

**Figure 10:**
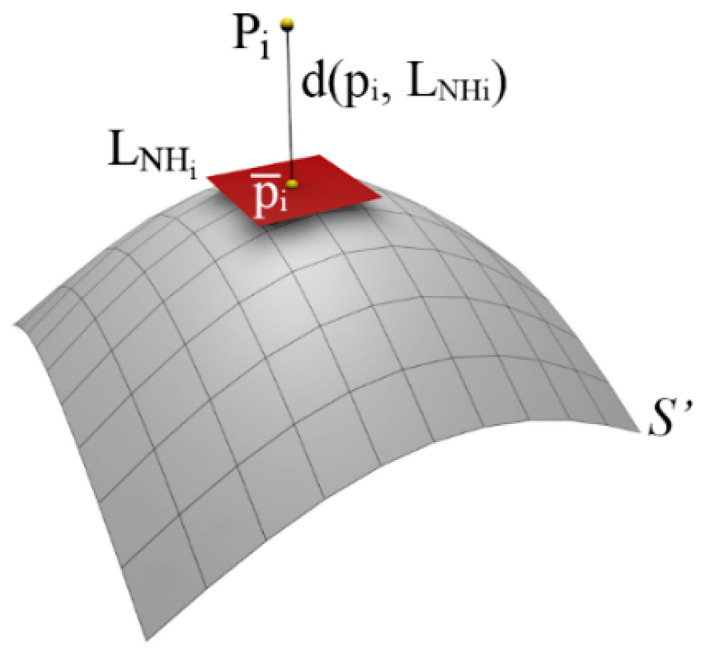
Local surface approximation and error computation as the distance from $p_{i}$ to $L_{NH_{i}}$.

For each point $p_{i}\in S$, the geometric error $d\left(p_{i},S^{\prime }\right)$, is defined as the Euclidean distance between the sampled point $p_{i}$ and its projection point $\bar{p_{i}}$ on the simplified surface approximation $S^{\prime }$. Since our method is mesh-free, we approximate the simplified surface $S^{\prime }$ using a least squares plane (LSP). To estimate the LSP, we select a set of neighboring points $NH_{i}$ in $P^{\prime }$ closest to $p_{i}$, using a Kd-tree data structure, and perform a PCA to obtain a regression plane ($L_{NH_{i}}$), which represents the local approximation $S^{\prime }$, i.e., $d\left(p_{i},S^{\prime }\right)≅d(p_{i},L_{NH_{i}})$ (Figure 10).

Table 1 shows the test models with the original sizes and the sampled points with different sampling rates (the value shown is the arithmetic average of the number of points resulting from the different methods for each simplification rate).

**Figure 11:**
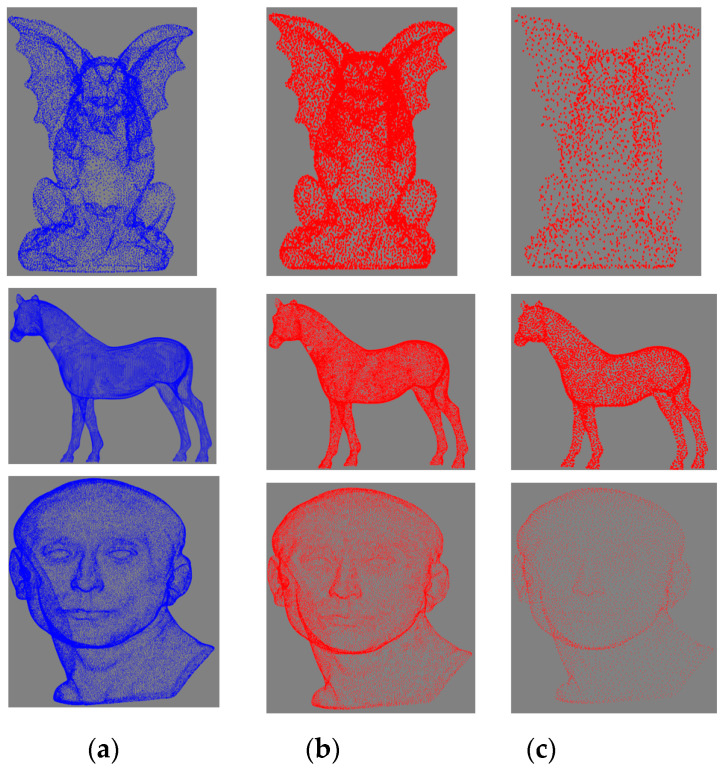
The Gargoyle, Horse, and Nicolo models: (**a**) original models; (**b**) models simplified at 50%; (**c**) models simplified at 10%.

Figure 11 shows the Gargoyle, Horse, and Nicolo models, as examples of Table 1; the originals are shown in the left column, the models simplified at 5% are shown in the middle column, and the models simplified at 50% are shown in the right column.

Table 2 shows the different values of the parameter δ for different simplification rates; we can appreciate how the variation of δ does not clarify the relationship between the number of points to be simplified and its values in the table. This indicates that the algorithm is sensitive when its values change between different simplification rates, showing a weakness of the algorithm, which can be improved if the parameter δ can be related to the density and distance between the points of the cloud to be simplified.

Table 3 shows the quantitative comparison between our method and the state-of-the-art methods. Table 3 shows four simplification rates, i.e., 5%, 10%, 20%, and 50%. All five methods reduce the original number of points to a similar number of simplified points. Our method provides the most accurate simplification result of the five algorithms with respect to the average error metric $\Delta_{\mathrm{avg}}$. However, considering the maximum error metric $\Delta_{\max}$, the Poisson disk mesh-based method is the best, closely followed by our method.

As shown in Table 3, the CV and PSD methods produce similar results in terms of average surface error. The PSD method achieved relatively better results in terms of maximum surface error; however, a mesh structure must be used in the simplification. There are some practical applications where only the 3D coordinate information is available, which limits the applicability of the PSD sampling method. The SGR method and our SDBS method achieved the best results in terms of average surface error, but the SDBS outperforms all other methods.

We compared the SDBS method with the other methods in accuracy and running time. Table 4 shows the running time and the number of preserved points of the proposed approach compared to six state-of-the-art methods. We simplified all the point clouds at a similar simplification rate with all the algorithms. We ran each method 10 times on each point cloud, and the average execution time is shown in Table 4. The programming language is also shown. It is worth noting that the simplification rate of our method is the lowest in the study (the Bunny model was simplified from 35,945 points to 4517 points, and the Elephant model was simplified from 24,955 points to 2154). The SDBS keeps the balance between the sharp features and the point density in the data set.

### 5.3. Visual Comparison

**Figure 12:**
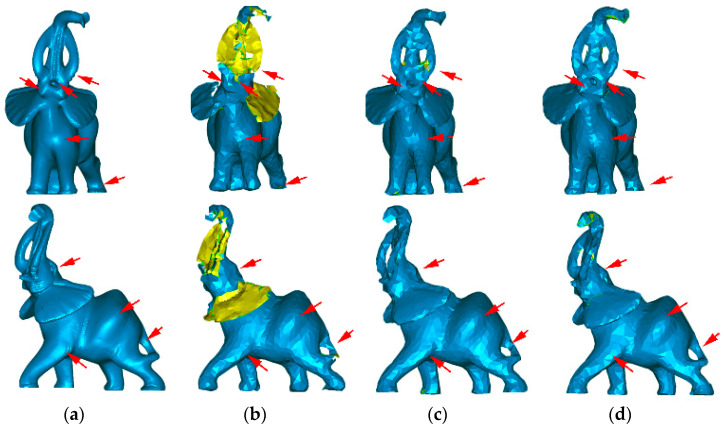
Effect on the lighting and density in (**a**) the original Elephant model when (**b**) the point coordinates are not included, (**c**) the normal coordinates are not included, and (**d**) all three features are included. The arrows show some of the lighting zones.

**Figure 13:**
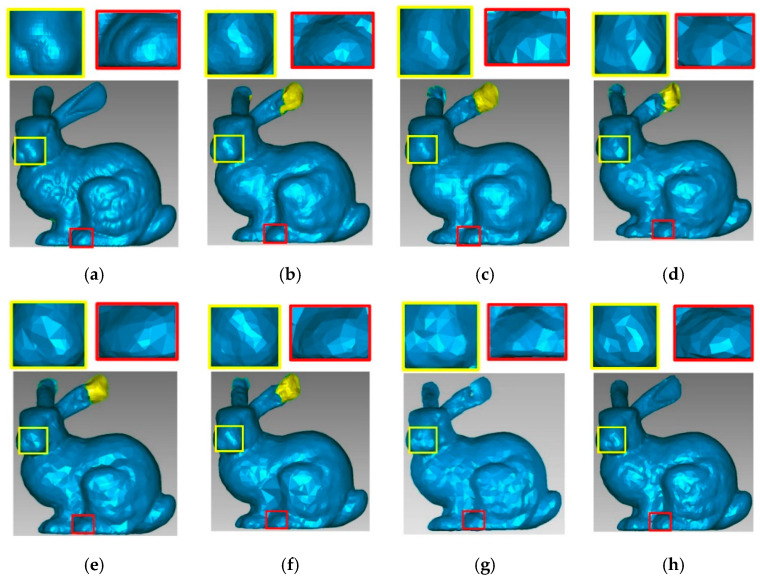
Point cloud simplification of the Bunny model. (**a**) The original data set, number of points = 35,947; (**b**) HCS method, number of points = 4644; (**c**) GRID method, number of points = 4562; (**d**) WLOP method, number of points = 4572; (**e**) FRGR method, number of points = 4638; (**f**) FPUC method, number of points = 4644; (**g**) DFPSA method, number of points = 4566; (**h**) proposed SDBS method, number of points = 4517. The image (g) is taken from [7].

**Figure 14:**
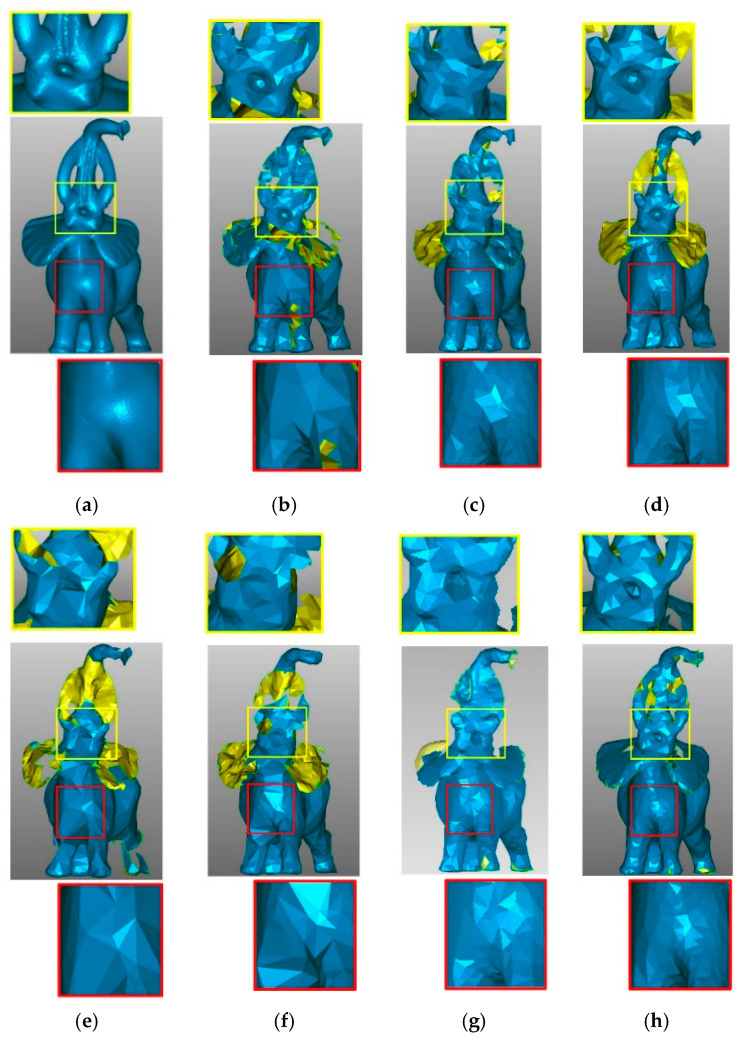
Point cloud simplification by different algorithms for the Elephant model. (**a**) The original data set, number of points = 24,955; (**b**) HCS method, number of points = 2184; (**c**) GRID method, number of points = 2684; (**d**) WLOP method, number of points = 2438; (**e**) FRGR method, number of points = 2164; (**f**) FPUC method, number of points = 2165; (**g**) DFPSA method, number of points = 2872; (**h**) proposed SDBS method, number of points = 2154. The image (g) is taken from [7].

To validate our method with respect to the visual quality of its results, we performed two experiments. The first experiment shows how the point cloud is affected in two scenarios: (1) when the normal coordinates are excluded from the feature vector and (2) when the coordinates of the point are excluded (Figure 12). The second experiment compares our results with different state-of-the-art methods (Figure 13 and Figure 14). For rendering purposes, our point clouds were meshed using the Geomagic Studio software.

Figure 12b shows the result of simplifying the elephant using only the normal and curvature, excluding the point coordinates from the feature vector of each point. Compared with the original model (Figure 12a), the simplification has overdecimated some areas (ears, tusk, and tube), producing holes in the reconstructed model. On the other hand, the lighting in the simplified model mimics the original one (red arrows). Figure 12c shows the simplification results for the Elephant model using the point coordinates and curvature, excluding the normal from the feature vector of each point. Compared with the original, the point density is maintained, producing a better reconstruction of the model surface, but the lighting of the simplification does not improve, as shown in Figure 12b (see highlighted details). Finally, Figure 12d shows the simplification results for the elephant using the normal, the point coordinates, and the surface variation (curvature). The combination of features improves the results, as shown in the details in the lighting and the preservation of details such as the elephant eye.

To compare visually the results of the studied algorithms, we simplified the models to approximately the same number of points with all methods. Figure 13 shows the simplified results of the application of different algorithms to the Bunny data set. Figure 13b,c,e–g shows how more points are retained in curved parts, while fewer points are kept in smooth parts. The simplification result of Figure 13d is uniform. All the methods present good reconstruction results but cannot reconstruct narrow features such as ears, except for the DFPSA method, which shows only a small hole. The proposed method (Figure 13h) retains the most relevant features and details of the model, and the reconstruction does not present the problems observed with the other algorithms. The zoomed regions (nose commissure and paw) highlight how our approach better preserves geometric details of the original point cloud compared to previous methods, even when the simplification rate of our method is lower than the others.

Figure 14 shows the simplification result for the Elephant data set with a high simplification rate. Figure 14c,d,g shows how the GRID, WLOP, and DFPSA simplification methods preserve few points in smooth regions and more points in feature regions such as legs, ears, trunk, and tusks. The HCS, FRGR, and FPUC simplification methods, as shown in Figure 14b,e,f, present problems in retaining the global structure of the respective point clouds. Our method also preserves more points in feature areas, but it distributes the points evenly in smooth regions. Due to the high simplification rate, all algorithms present failures, but our method is the best in preserving the overall structure of the data set, as shown in the zoomed regions (mouth and chest), even when the simplification rate of our method is lower than the others.

## 6. Conclusions and Future Work

In this paper, we have presented a new method for point cloud simplification based on dictionary learning and sparse coding. The proposed method preserves the sharp features and produces evenly distributed points. Our method uses the normal vector, curvature, and the position of the points as a component of a feature vector. The feature vectors of all points of the cloud are the input for a dictionary learning and sparse coding process for saliency detection. We use the sparse representation of a signal to establish when a point is salient or not for the entire point cloud; i.e., points are considered salient if their feature vectors are reconstructed with many atoms from the dictionary, while points are not considered salient if the feature vectors are reconstructed with few atoms. The simplification is guided by global saliency using the sparse vectors resulting from the sparse coding process; we use its sparsity as an adaptive simplification ratio in different regions. The proposed method produces low simplification rates in salient regions (borders, corners, high curvatures, valleys) and high simplification rates in relatively planar regions while maintaining an appropriate density through an even distribution of points.

The robustness and efficiency of our approach are demonstrated by some experimental results that show that our method reduces the size of point clouds and retains the shape features without creating surface holes. Finally, the proposed method is compared with different state-of-the-art approaches, producing good simplification results and outperforming competing methods. As future work, we propose examining ways to automatically determine the choice of the regularization parameter λ and the size of the dictionary, S. Another future work is the mathematical demonstration of the interpretation when a point is considered salient or not salient and how to relate the δ directly with the number of points to be simplified.

## Figures and Tables

**Table 1:** Test models with the original number of points and the sampling results at different simplification rates.

Models	Original Points	Sampled Points 5%	Sampled Points 10%	Sampled Points 20%	Sampled Points 50%
Bunny	35,947	1797	3610	7186	17,976
Elephant	24,955	1246	2489	4991	12,478
Gargoyle	25,038	1253	2496	5008	12,522
Horse	48,485	2428	4872	9693	24,247
Max Plank	50,112	2459	4892	9826	24,569
Nicolo	50,419	2519	5053	10,082	25,213
Fandisk	25,894	1249	2480	4974	12,437

**Table 2:** Values of δ used for different models, when they were simplified at 5%, 10%, 20% and 50%.

Models	δ Value Sampled 5%	δ Value Sampled 10%	δ Value Sampled 20%	δ Value Sampled 50%
Bunny	0.00140	0.000968	0.000627	0.000348
Elephant	0.01233	0.008330	0.005700	0.003460
Gargoyle	0.08000	0.046700	0.030200	0.015000
Horse	0.00133	0.000900	0.000600	0.000333
Max Plank	0.15330	0.076700	0.040000	0.015670
Nicolo	0.02533	0.018000	0.001180	0.006533
Fandisk	0.00053	0.000365	0.000266	0.000176

**Table 3:** Simplification results, comparison at different sampling rates (SRs) (5%, 10%, 20% and 50%) using the $\Delta_{max}$ and $\Delta_{avg}$ metrics between the proposed method and the state-of-the-art methods.

	Mesh-Based		Point-Based							
	PSD		CV		FRGR		FPUC		SDBS	
SR 5%	$\Delta_{max}$	$\Delta_{avg}$	$\Delta_{max}$	$\Delta_{avg}$	$\Delta_{max}$	$\Delta_{avg}$	$\Delta_{max}$	$\Delta_{avg}$	$\Delta_{max}$	$\Delta_{avg}$
Bunny	**0.005065**	0.000535	0.012529	0.000786	0.010989	0.000781	0.023727	0.001267	0.006019	**0.000517**
Elephant	**0.029524**	0.004453	0.071016	0.006473	0.079300	0.007481	0.076502	0.006723	0.032534	**0.004307**
Gargoyle	**0.520473**	0.096518	1.920498	0.129355	2.191607	0.130839	1.334782	0.222911	0.653588	**0.090725**
Horse	**0.003544**	0.000343	0.008435	0.000487	0.009263	0.000490	0.017894	0.001056	0.004340	**0.000322**
Max Plank	**1.301519**	0.099681	3.702109	0.145190	2.802459	0.165132	4.725397	0.283958	1.618001	**0.087707**
Nicolo	**0.134143**	0.011021	0.370415	0.015898	0.331987	0.016816	0.292358	0.015014	0.149977	**0.010250**
Fandisk	**0.206912**	0.017887	1.251090	0.079158	0.441557	0.032766	0.627595	0.044314	0.215029	**0.016890**
SR 10%	$\Delta_{max}$	$\Delta_{avg}$	$\Delta_{max}$	$\Delta_{avg}$	$\Delta_{max}$	$\Delta_{avg}$	$\Delta_{max}$	$\Delta_{avg}$	$\Delta_{max}$	$\Delta_{avg}$
Bunny	**0.004430**	0.000308	0.008944	0.000426	0.007164	0.000416	0.012347	0.000496	0.005212	**0.000287**
Elephant	**0.022318**	0.002414	0.048641	0.003695	0.057984	0.003880	0.051137	0.003412	0.029524	**0.002312**
Gargoyle	**0.038192**	0.006216	1.283042	0.082879	2.162895	0.084856	0.968352	0.012522	0.054968	**0.005627**
Horse	**0.002506**	0.000190	0.006470	0.000261	0.005413	0.000245	0.008680	0.000369	0.003544	**0.000171**
Max Plank	**0.961236**	0.059349	2.856882	0.084547	1.798308	0.085617	4.404936	0.135728	1.240950	**0.049349**
Nicolo	**0.106050**	0.006580	0.246437	0.009023	0.280581	0.009329	0.177455	0.008315	0.116171	**0.005710**
Fandisk	**0.149206**	0.009414	1.240782	0.050776	0.319061	0.020614	0.432045	0.016975	0.154839	**0.009010**
SR 20%	$\Delta_{max}$	$\Delta_{avg}$	$\Delta_{max}$	$\Delta_{avg}$	$\Delta_{max}$	$\Delta_{avg}$	$\Delta_{max}$	$\Delta_{avg}$	$\Delta_{max}$	$\Delta_{avg}$
Bunny	**0.003009**	0.000183	0.008241	0.000223	0.005630	0.000226	0.006616	0.000227	0.003475	**0.000151**
Elephant	**0.017644**	0.001367	0.039453	0.001812	0.046601	0.002064	0.035288	0.001663	0.019328	**0.001171**
Gargoyle	**0.306220**	0.040987	0.924313	0.047888	2.148394	0.050902	0.603874	0.069340	0.467759	**0.033819**
Horse	**0.002046**	0.000109	0.004798	0.000134	0.004340	0.000132	0.005011	0.000139	0.002506	**0.000087**
Max Plank	**0.679693**	0.033580	2.000972	0.042923	1.301519	0.044349	1.359393	0.048901	0.961236	**0.024317**
Nicolo	**0.094854**	0.003902	0.217337	0.004568	0.189707	0.004759	0.157297	0.004514	**0.094854**	**0.003155**
Fandisk	**0.101366**	0.005030	1.240782	0.035560	0.242986	0.014598	0.411750	0.009057	0.130863	**0.004398**
SR 50%	$\Delta_{max}$	$\Delta_{avg}$	$\Delta_{max}$	$\Delta_{avg}$	$\Delta_{max}$	$\Delta_{avg}$	$\Delta_{max}$	$\Delta_{avg}$	$\Delta_{max}$	$\Delta_{avg}$
Bunny	**0.002128**	0.000094	0.006143	0.000072	0.003885	0.000077	0.002747	0.000067	0.002747	**0.000060**
Elephant	**0.006863**	0.000717	0.028450	0.000592	0.024952	0.000624	0.017644	0.000516	0.017644	**0.000526**
Gargoyle	**0.228243**	0.024144	0.653588	0.017368	1.436299	0.017210	0.368031	0.020228	0.250028	**0.012773**
Horse	**0.002046**	0.000054	0.003544	0.000043	0.002506	0.000041	0.004340	0.000038	**0.002046**	**0.000033**
Max Plank	**0.554970**	0.016812	1.359393	0.015499	0.877484	0.013778	0.784846	0.012912	**0.554970**	**0.009639**
Nicolo	**0.067072**	0.001986	0.142280	0.001626	0.106050	0.001638	0.067072	0.001366	**0.067072**	**0.001191**
Fandisk	**0.071676**	0.002220	1.237327	0.030615	0.155924	0.009728	0.383764	0.005845	0.092534	**0.001499**

**Table 4:** Comparison of simplification time and preserved number of points.

Method	Preserved Number of Points (Bunny)	Preserved Number of Points (Elephant)	Bunny Running Time (s)	Elephant Running Time (s)	Language
SDBS	4517	2154	21.223	15.186	MATLAB
DFPSA	4566	2872	56.156	26.220	---
FPUC	4644	2165	38.094	29.503	MATLAB
FRGR	4638	2164	9.5740	1.0030	MATLAB
WLOP	4572	2438	16.678	10.879	C/C++
GRID	4562	2154	0.6920	0.5170	C/C++
HCS	4644	2184	4.4590	3.1470	C/C++

## Data Availability

Not applicable.
